# Supplementary material for: CTCF is a barrier for 2C-like reprogramming
Source: Nat Commun. 2021 Aug 11;12:4856. doi: 10.1038/s41467-021-25072-x (PMC8358036; doi:10.1038/s41467-021-25072-x)
Supplement: Supplementary file 1 — Supplementary Information [file 41467_2021_25072_MOESM1_ESM.pdf]

Supplementary Information:

Supplementary information includes 11 Supplementary Figures and 1 Supplementary Table.

CTCF is a Barrier for 2C-like Reprogramming

Teresa Olbrich et al,

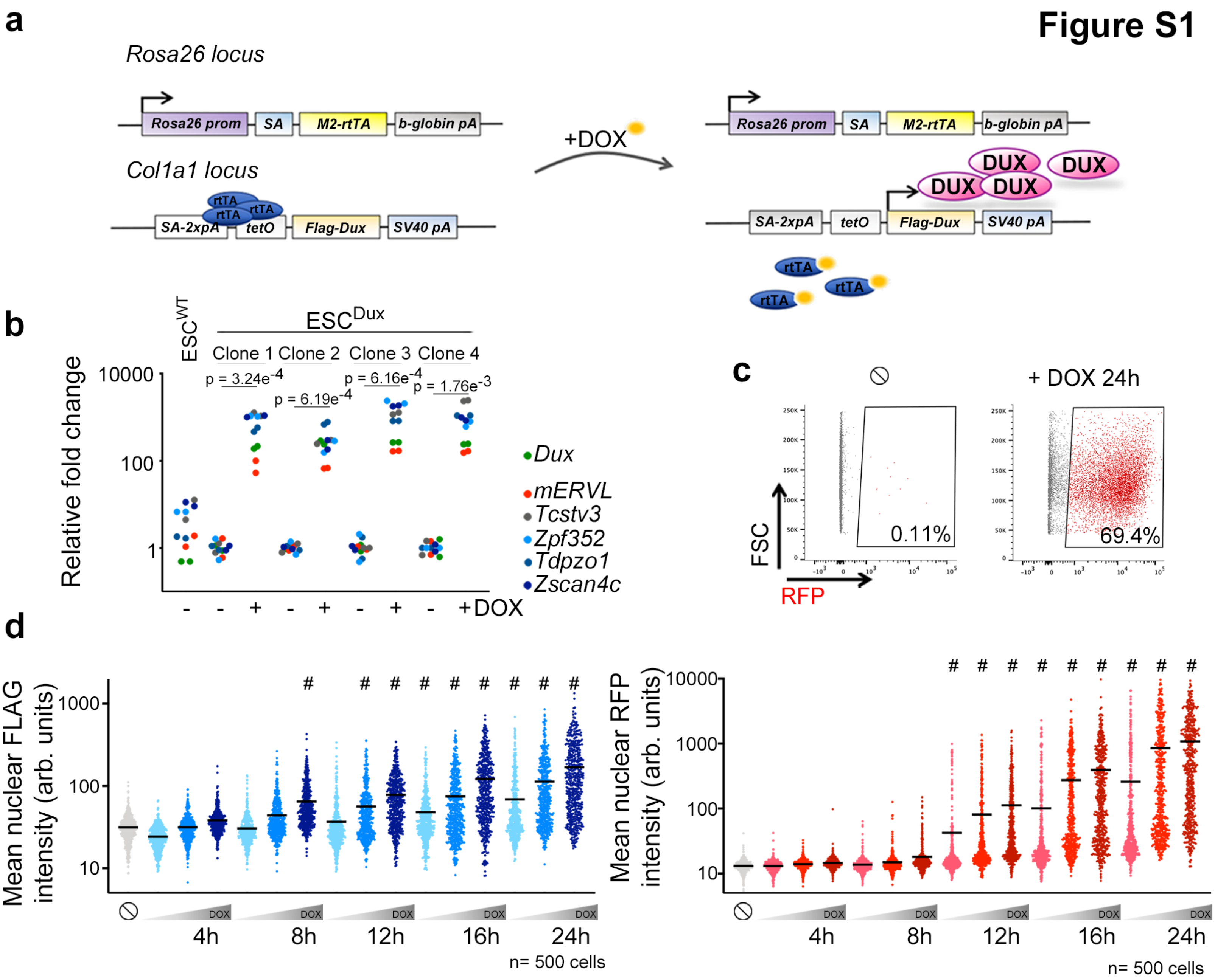

Figure S1
a
Rosa26 locus
Rosa26 prom
SA
M2-rtTA
b-globin pA
Col1a1 locus
rtTA
SA-2xpA
tetO
Flag-Dux
SV40 pA
+DOX
DUX
b
ESC^WT
ESC^Dux
Clone 1 Clone 2 Clone 3 Clone 4
p = 3.24e-4
p = 6.19e-4
p = 6.16e-4
p = 1.76e-3
Relative fold change
10000
100
1
Dux
mERVL
Tcstv3
Zpf352
Tdpzo1
Zscan4c
+DOX
c
+ DOX 24h
FSC
RFP
0.11%
69.4%
d
Mean nuclear FLAG intensity (arb. units)
Mean nuclear RFP intensity (arb. units)
DOX
4h
8h
12h
16h
24h
n= 500 cells

Supplementary Fig. 1: ESC^{DUX} recapitulated all 2C-like features upon DOX-induction. a, Schematic representation of the two-allele system used to generate ESC^{DUX}. DUX cDNA was placed under the control of a tet-responsible sequence (tetO) at the *Col1a1* locus while rtTA is expressed from the *Rosa26* locus[11]. **b,** Graph showing the relative fold change (log10) expression of six 2C associated genes in ESC^{DUX} untreated or treated with 300ng/ml DOX for 24 hours. Four ESC clones were tested but two were selected for further downstream analyses. Real-time PCR reactions were performed by duplicate and one representative experiment is shown. GAPDH expression was used to normalize gene expression. All genes were considered as a group for statistical analysis. p values are shown from two-tailed paired *t*-tests. **c,** Flow cytometry analysis performed in untreated or DOX-treated (300ng/ml) LTR-RFP reporter ESC^{DUX} for 24 hours. Percentages of RFP^{+} are included within the plots. ∅=No treatment. **d,** HTI quantification of FLAG (left plot) and RFP (right plot) expression in untreated or dox-treated for the indicated times with different DOX concentrations in *LTR-RFP* reporter ESC^{DUX}. Center lines indicate mean values. In (**c**, **d**), one representative experiment is shown but at least two independent experiments using multiple clones were performed. n=500; # = $p<0.001$ in two-tailed unpaired *t*-tests when compared to untreated ESC. DOX concentrations used: 150, 300 and 600 ng/ml.

Figure S2

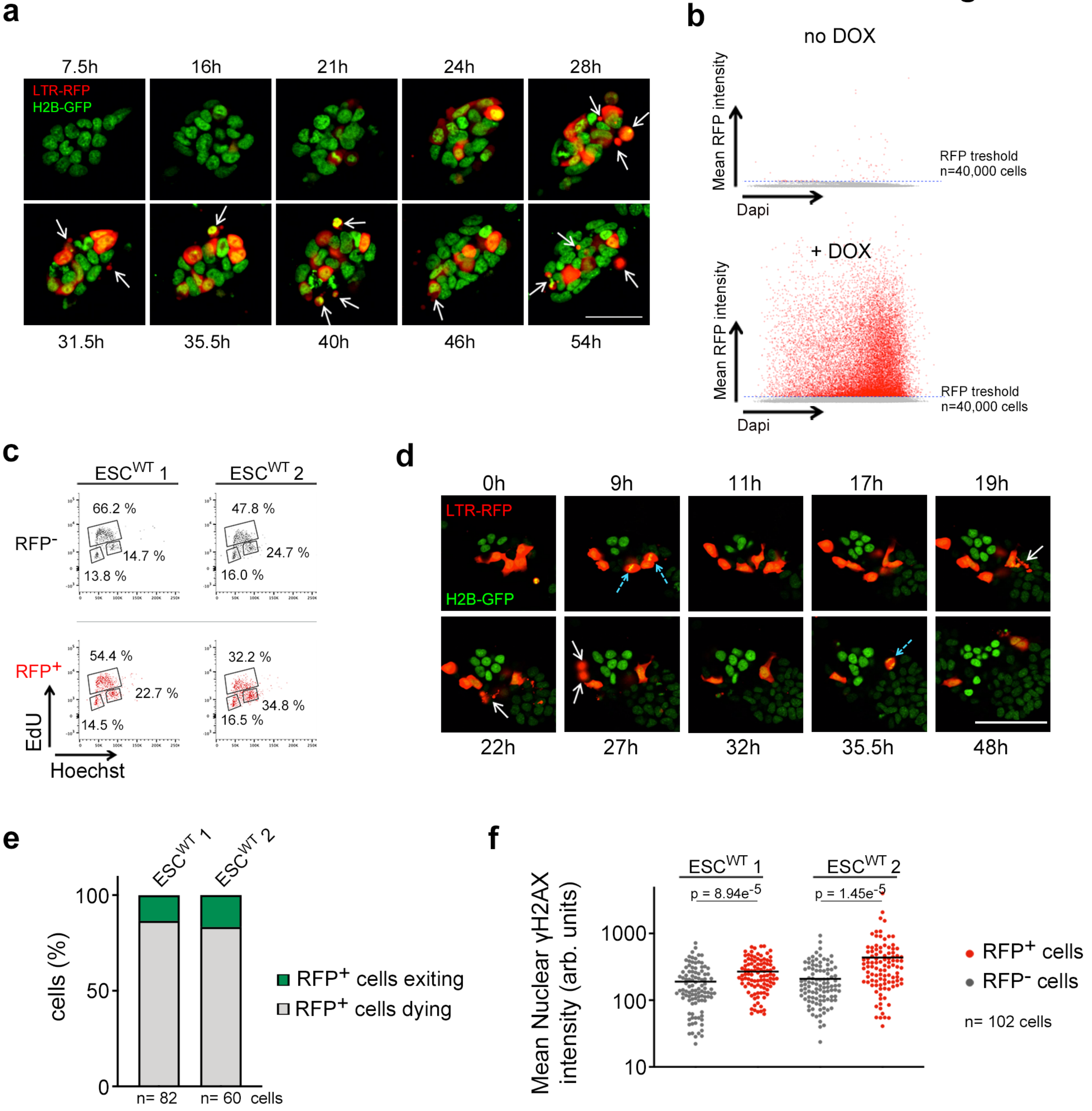
a
7.5h
16h
21h
24h
28h
LTR-RFP
H2B-GFP
31.5h
35.5h
40h
46h
54h
b
no DOX
Mean RFP intensity
Dapi
RFP treshold
n=40,000 cells
+ DOX
Mean RFP intensity
Dapi
RFP treshold
n=40,000 cells
c
ESCWT 1
ESCWT 2
RFP-
66.2 %
14.7 %
13.8 %
47.8 %
24.7 %
16.0 %
RFP+
54.4 %
22.7 %
14.5 %
32.2 %
34.8 %
16.5 %
EdU
Hoechst
d
0h
9h
11h
17h
19h
LTR-RFP
H2B-GFP
22h
27h
32h
35.5h
48h
e
ESCWT 1
ESCWT 2
cells (%)
100
50
0
n= 82
n= 60 cells
RFP+ cells exiting
RFP+ cells dying
f
ESCWT 1
ESCWT 2
p = 8.94e-5
p = 1.45e-5
Mean Nuclear γH2AX intensity (arb. units)
1000
100
10
RFP+ cells
RFP- cells
n= 102 cells

Supplementary Fig. 2: Endogenous 2C-like cells showed G2 arrest and increased γH2AX. a, Representative images obtained from a time lapse experiment where LTR-RFP reporter ESC^{DUX} expressing H2B-GFP were treated with 300ng/ml DOX and imaged at the indicated timepoints. Scale bar, 100 μm. White arrows show dying RFP^{+} ESC. Two independent experiments were performed with two ESC clones with similar results but one representative is shown. **b,** Dot plots showing cell distribution based on DNA content and RFP expression in LTR-RFP reporter ESC^{DUX} treated with 300ng/ml DOX for 24 hours. **c,** Flow cytometry analysis of the cell cycle distribution in two wild type LTR-RFP reporter ESC lines (R1 and G4 ESC). Cells were split in RFP^{-} or RFP^{+} subpopulations and percentages for each phase of the cells cycle are included. One representative experiment is shown but at least two independent experiments were performed. **d,** Representative images obtained from a live cell time lapse experiment where LTR-RFP reporter R1 ESC expressing H2B-GFP were followed over time. Indicated times showed the time when recording started. Two independent wild type LTR-RFP reporter ESC lines (R1 and G4) were imaged in two independent experiments but only one representative ESC line (R1) is shown. White arrows showed RFP^{+} ESC undergoing cell death. Blue dashed arrows showed RFP^{+} ESC undergoing cell division. Scale bar, 100 μm. **e,** Plot showing the percentage of endogenous cycling 2C-like cells in two wild-type ESC lines that successfully exit from the totipotent-like state and return to pluripotency or undergo cell death. Quantification was performed by following the fate of a total of 82 and 60 2C-like cells per cell line in the time-lapse experiments from (**d**). **f,** HTI quantification of γH2AX in two wild type LTR-RFP reporter ESC lines (R1 and G4 ESC). Cells were split in RFP^{-} and RFP^{+} subpopulations. Center lines indicate mean values. n=102; p values are

shown from two-tailed unpaired *t*-tests. One representative experiment is shown but two independent experiments were performed.

Figure S3

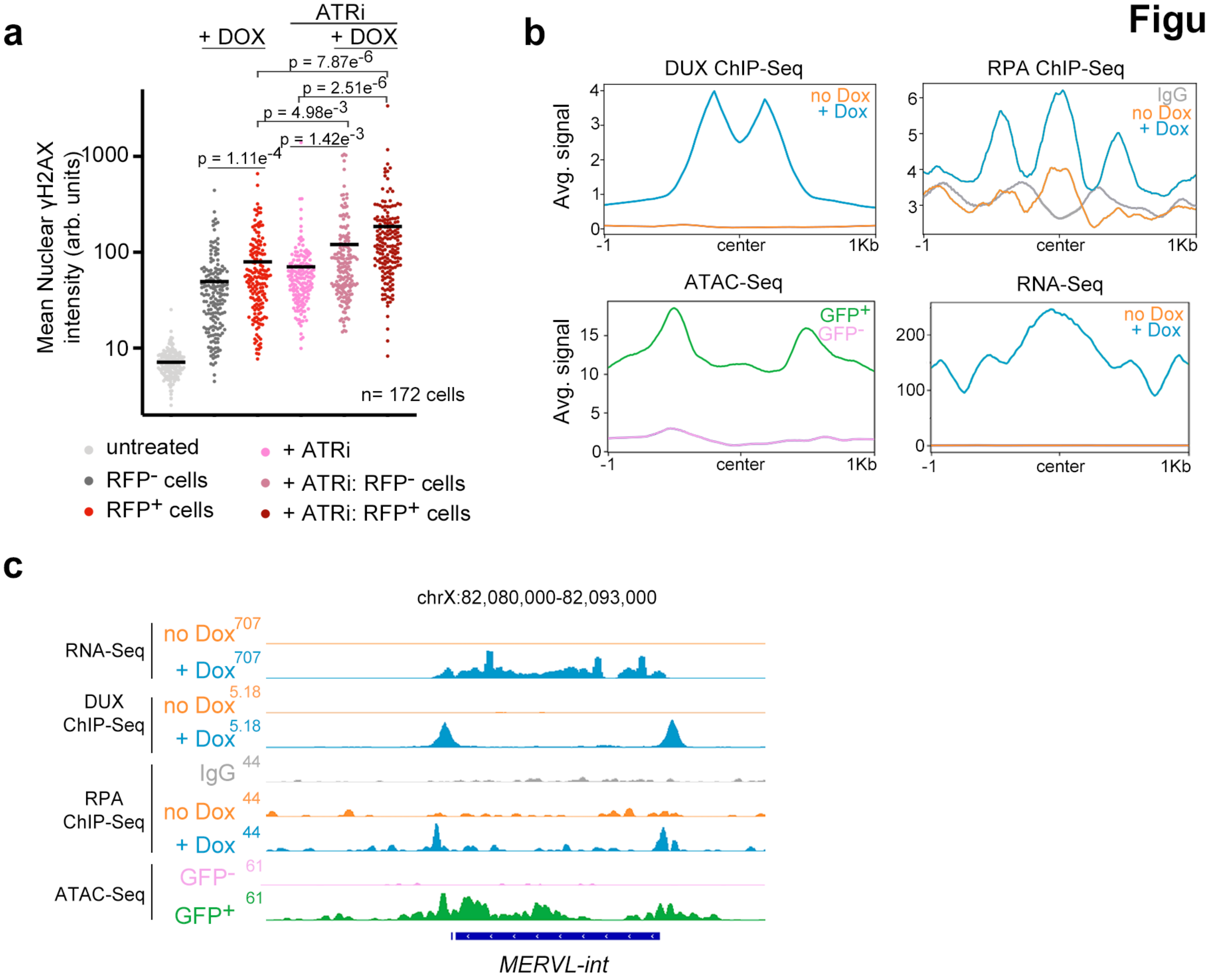
a
+ DOX
ATRi
+ DOX
p = 7.87e-6
p = 2.51e-6
p = 4.98e-3
p = 1.42e-3
p = 1.11e-4
Mean Nuclear γH2AX intensity (arb. units)
1000
100
10
n= 172 cells
untreated
RFP- cells
RFP+ cells
+ ATRi
+ ATRi: RFP- cells
+ ATRi: RFP+ cells
b
DUX ChIP-Seq
RPA ChIP-Seq
Avg. signal
no Dox
+ Dox
IgG
-1
center
1Kb
ATAC-Seq
RNA-Seq
GFP+
GFP-
c
chrX:82,080,000-82,093,000
RNA-Seq
DUX ChIP-Seq
RPA ChIP-Seq
ATAC-Seq
MERVL-int

Supplementary Fig. 3: Transcription-dependent RS induced by DUX expression. a, HTI quantification of γH2AX in LTR-RFP reporter ESC^{DUX} treated with 300ng/ml DOX and/or with 1μM ATR inhibitor (ATRi) for 24 hours. Center lines indicate mean values. Cells were split in RFP^- or RFP^+ and data is representative of at least two independent experiments performed in two different clones. n=172; p values are shown from two-tailed unpaired *t*-tests. Only statistics for relevant comparisons are shown. **b,** Average read density plots (RPKM) showing RPA, DUX occupancy and RNAseq read counts from untreated or 300ng/ml or 1μg/ml DOX-treated DUX expressing ESC (our data and [5]). ATACseq signal is shown from GFP^- or GFP^+ cells sorted 1μg/ml DOX-treated LTR-GFP reporter ESC^{DUX} [5]. Plots were generated using the 1000 MERVL genomic features with the highest level of bound DUX[5]. **c,** Genome browser tracks showing RPA, DUX occupancy, RNAseq read counts and ATACseq signal in the region surrounding a representative MERVL unit.

Figure S4

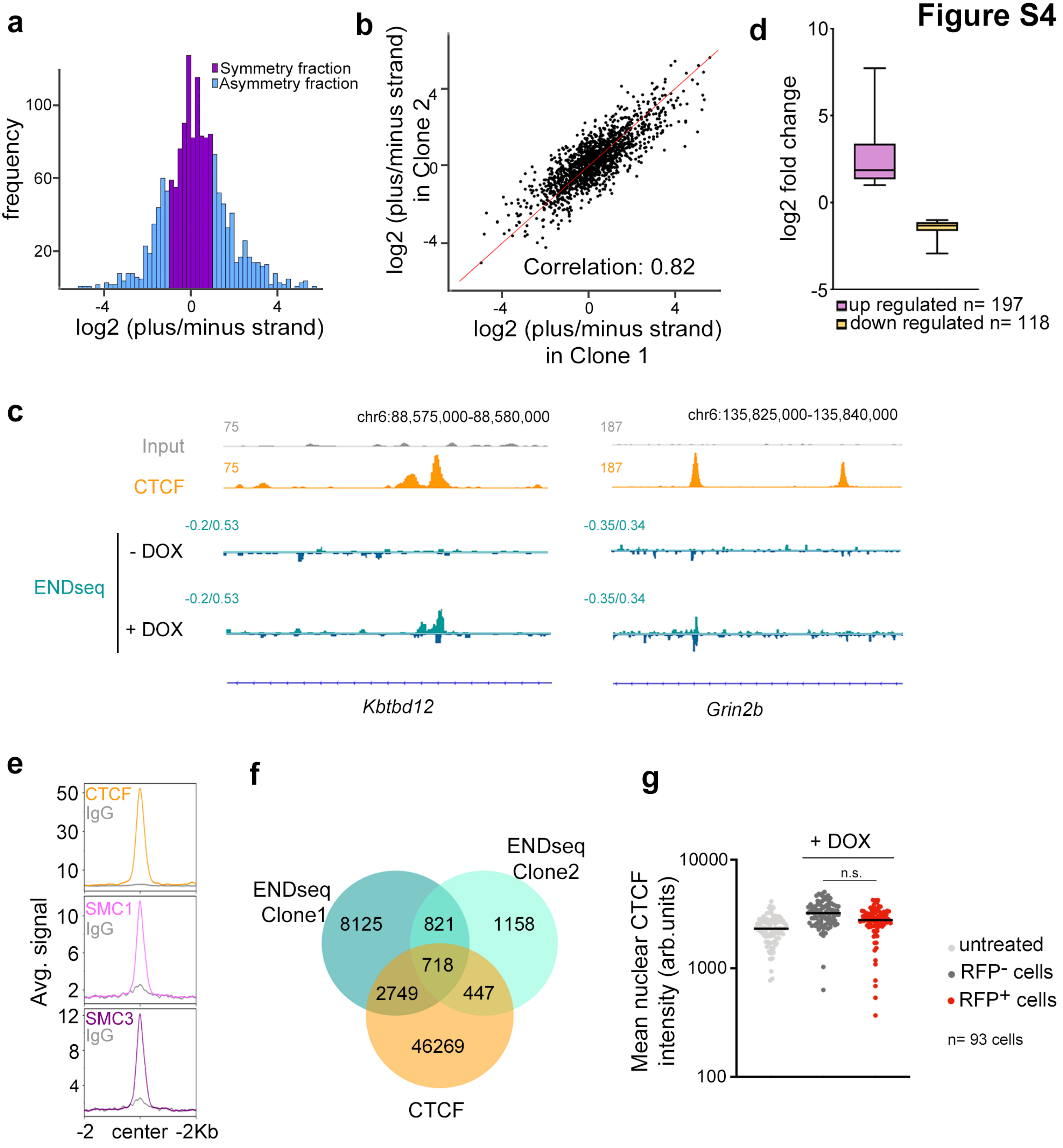
a
Symmetry fraction
Asymmetry fraction
frequency
log2 (plus/minus strand)
b
log2 (plus/minus strand) in Clone 2
log2 (plus/minus strand) in Clone 1
Correlation: 0.82
d
log2 fold change
up regulated n= 197
down regulated n= 118
c
chr6:88,575,000-88,580,000
chr6:135,825,000-135,840,000
Input
CTCF
ENDseq
- DOX
+ DOX
-0.2/0.53
-0.35/0.34
Kbtbd12
Grin2b
e
CTCF
IgG
SMC1
SMC3
Avg. signal
-2 center -2Kb
f
ENDseq Clone1
ENDseq Clone2
CTCF
8125
821
1158
718
2749
447
46269
g
+ DOX
n.s.
Mean nuclear CTCF intensity (arb.units)
untreated
RFP- cells
RFP+ cells
n= 93 cells

Supplementary Fig. 4: CTCF is enriched in END-seq sites.

a, Histogram plot showing the frequency of single (asymmetric fraction) or double (symmetric fraction) strand breaks observed within the END-seq peaks. Ratio between the signal intensity per strand was used to define symmetric or asymmetric END-seq peaks. **b,** Plot showing the level of correlation between the type of DNA lesions (single or double strand breaks) observed in two independent ESC^{DUX} clones. Pearson correlation value is shown. **c,** Genome browser tracks showing END-seq signal in untreated and 300ng/ml DOX-treated ESC^{DUX} at the indicated genome location. One representative single (left panel) or double (right panel) end break is shown. Input (IgG) is shown as a background reference control. **d,** Box and whisker plot showing the relative fold change (log2) in genes differentially expressed (a total of 314 genes) between wild-type and DUX-expressing ESC located near END-seq sites (Supplementary Table 5). Center line indicates the median, box extends from the 25th to 75th percentiles and whiskers show Min to Max values. Data was obtained from[5]. **e,** Plots showing CTCF[20], SMC1 and SMC3[21] enrichment at the set of 1539 END-seq sites identified in DOX-treated ESC^{DUX}. Input (IgG) is also shown as a background reference control. **f,** Venn diagram showing the number of END-seq and CTCF overlapping peaks between the two ESC^{DUX} clones analyzed. Peak calling for CTCF binding sites in ESC was obtained from[20]. **g,** HTI quantification of CTCF in untreated or 600ng/ml DOX-treated ESC^{DUX} for 24 hours. Cells were split into RFP^+ or RFP^- subpopulations. Center lines indicate mean values. n=93; n.s.=non-significant in two-tailed unpaired *t*-test. Representative data from one ESC^{DUX} clone is shown but two independent clones were analyzed.

a b **Figure S5**

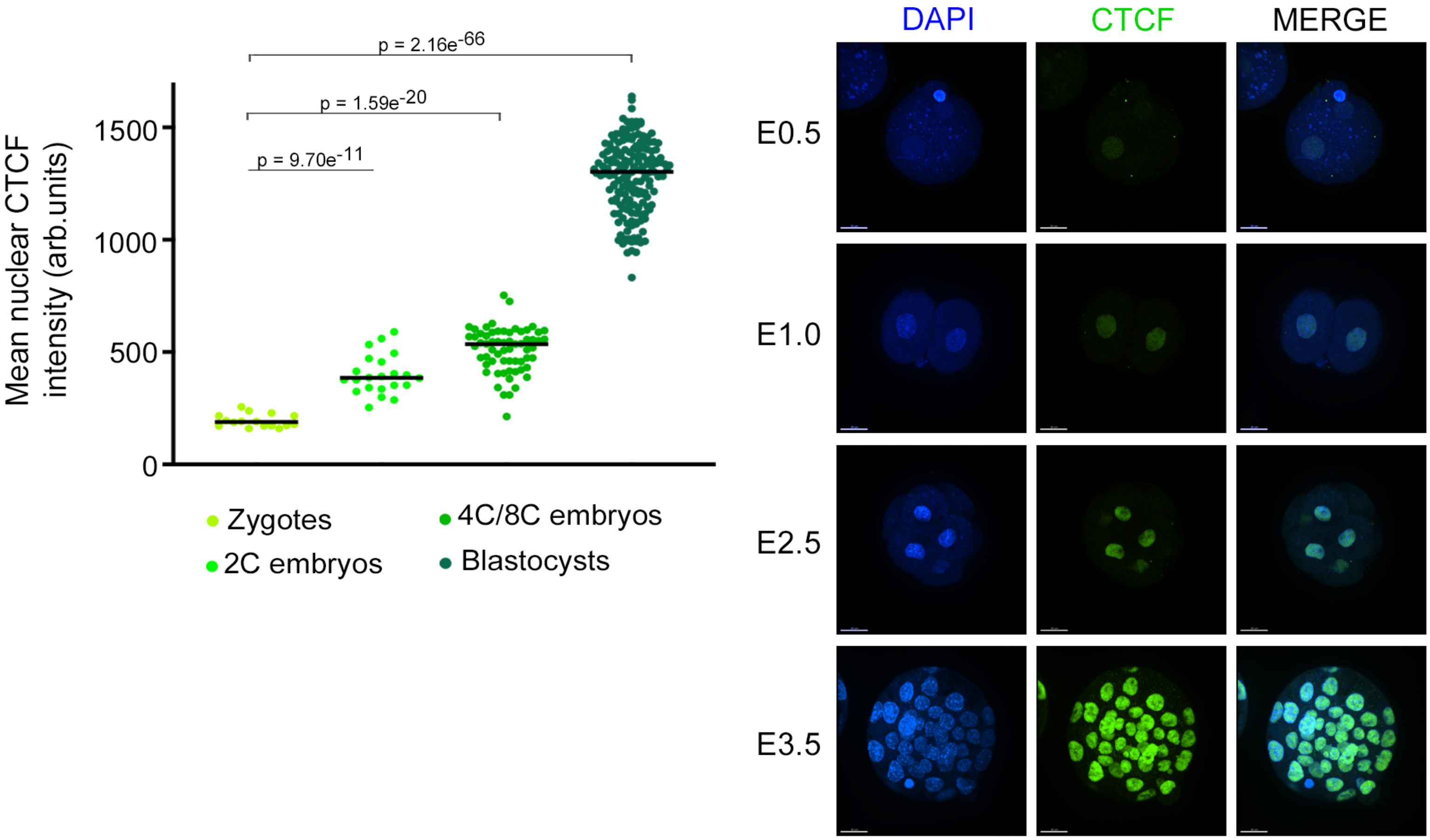
p = 2.16e-66
p = 1.59e-20
p = 9.70e-11
Mean nuclear CTCF intensity (arb.units)
1500
1000
500
0
Zygotes
2C embryos
4C/8C embryos
Blastocysts
DAPI
CTCF
MERGE
E0.5
E1.0
E2.5
E3.5

Supplementary Fig. 5: CTCF expression levels increase during embryonic development. a, Graph showing CTCF mean nuclear intensity in mouse embryos at different developmental stages, Zygotes (E0.5, 8 embryos), 2C (E1.0, 11 embryos), 4C/8C (E2.5, 14 embryos) and blastocysts (E3.5, 7 embryos). p values are shown from two-tailed unpaired *t*-tests. **b,** Representative images from the mouse embryos described in (**a**). Scale bar, 20μm.

Figure S6

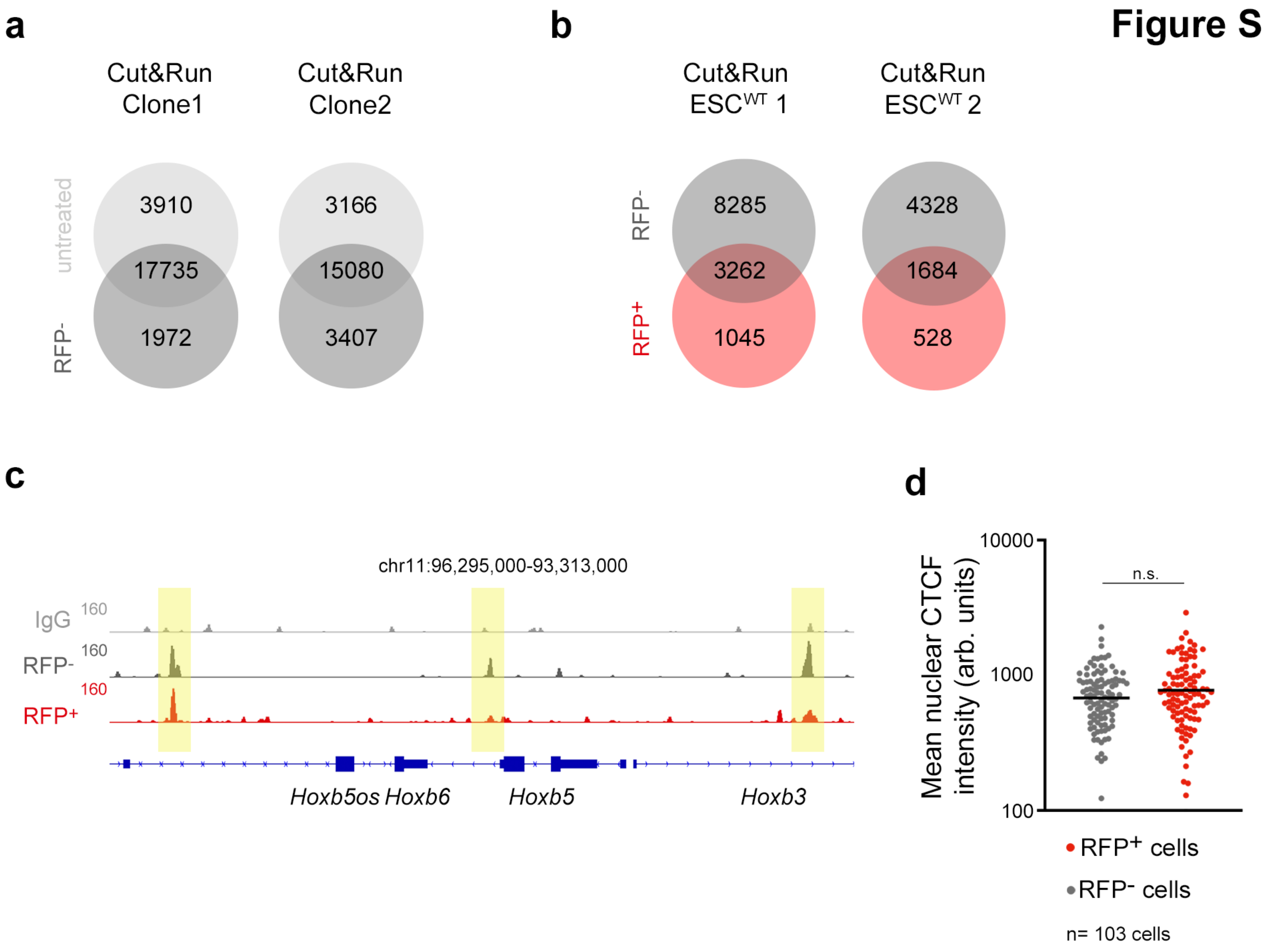
a
Cut&Run Clone1
Cut&Run Clone2
untreated
RFP-
3910
17735
1972
3166
15080
3407
b
Cut&Run ESC^WT 1
Cut&Run ESC^WT 2
RFP-
RFP+
8285
3262
1045
4328
1684
528
c
chr11:96,295,000-93,313,000
IgG 160
RFP- 160
RFP+ 160
Hoxb5os Hoxb6
Hoxb5
Hoxb3
d
Mean nuclear CTCF intensity (arb. units)
10000
1000
100
n.s.
RFP+ cells
RFP- cells
n= 103 cells

Supplementary Fig. 6: Endogenous 2C-like cells showed a reorganized CTCF binding landscape. **a,** Venn diagram showing the number of CTCF peaks detected in untreated and RFP^- sorted cells from two independent DOX-treated ESC^{DUX} clones. **b,** Venn diagram showing the number of CTCF peaks detected in RFP^- and RFP^+ sorted cells from two wild type ESC lines. **c,** Genome browser tracks showing CTCF occupancy at the indicated genome location in one representative wild-type cell line. Input (IgG) is shown as a background reference control. **d,** HTI quantification of CTCF in wild-type LTR-RFP reporter ESC. Cells were split into RFP^+ or RFP^- subpopulations. Center lines indicate mean values. n=103; n.s.=non-significant in two-tailed unpaired *t*-test. Representative data from one wild-type ESC clone is shown but two independent clones were analyzed.

Figure S7

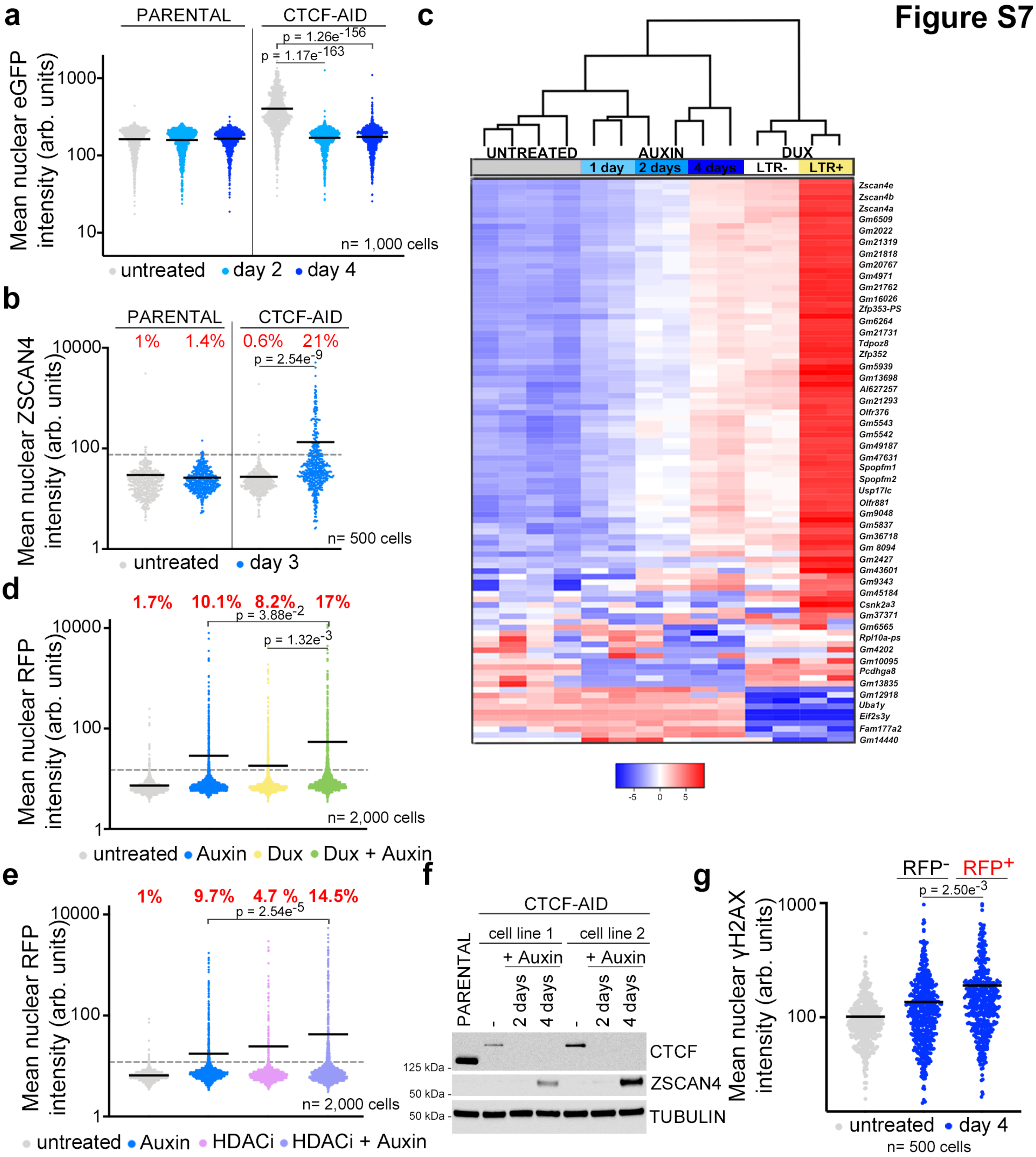

a
PARENTAL
CTCF-AID
p = 1.26e-156
p = 1.17e-163
Mean nuclear eGFP intensity (arb. units)
1000
100
10
n= 1,000 cells
untreated
day 2
day 4
b
PARENTAL
CTCF-AID
1%
1.4%
0.6%
21%
p = 2.54e-9
Mean nuclear ZSCAN4 intensity (arb. units)
10000
100
1
n= 500 cells
untreated
day 3
c
UNTREATED
AUXIN
DUX
1 day
2 days
4 days
LTR-
LTR+
Zscan4e
Zscan4b
Zscan4a
Gm6509
Gm2022
Gm21319
Gm21818
Gm20767
Gm4971
Gm21762
Gm16026
Zfp353-PS
Gm6264
Gm21731
Tdpoz8
Zfp352
Gm5939
Gm13698
AI627257
Gm21293
Olfr376
Gm5543
Gm5542
Gm49187
Gm47631
Spopfm1
Spopfm2
Usp17lc
Olfr881
Gm9048
Gm5837
Gm36718
Gm 8094
Gm2427
Gm43601
Gm9343
Gm45184
Csnk2a3
Gm37371
Gm6565
Rpl10a-ps
Gm4202
Gm10095
Pcdhga8
Gm13835
Gm12918
Uba1y
Eif2s3y
Fam177a2
Gm14440
-5
0
5
d
1.7%
10.1%
8.2%
17%
p = 3.88e-2
p = 1.32e-3
Mean nuclear RFP intensity (arb. units)
10000
100
1
n= 2,000 cells
untreated
Auxin
Dux
Dux + Auxin
e
1%
9.7%
4.7 %
14.5%
p = 2.54e-5
Mean nuclear RFP intensity (arb. units)
10000
100
1
n= 2,000 cells
untreated
Auxin
HDACi
HDACi + Auxin
f
CTCF-AID
cell line 1
cell line 2
+ Auxin
+ Auxin
PARENTAL
-
2 days
4 days
-
2 days
4 days
125 kDa
50 kDa
50 kDa
CTCF
ZSCAN4
TUBULIN
g
RFP-
RFP+
p = 2.50e-3
Mean nuclear γH2AX intensity (arb. units)
1000
100
untreated
day 4
n= 500 cells

Supplementary Fig. 7: CTCF depletion induces spontaneous 2C-like conversion in ESC. a, HTI quantification of GFP in untreated or auxin-treated for two and four days parental and ESC$^{CTCF\text{-}AID}$. Center lines indicate mean values. n=1000; p values are shown from two-tailed unpaired *t*-tests. **b,** HTI quantification of ZSCAN4$^+$ cells in untreated or auxin-treated for three days parental and ESC$^{CTCF\text{-}AID}$. Center lines indicate mean values. Percentages of ZSCAN4$^+$ cells above the threshold are indicated. n=500; p values are shown from two-tailed unpaired *t*-tests. **c,** Heatmap generated from RNAseq data from untreated or auxin-treated ESC$^{CTCF\text{-}AID}$ at different time points[23] together with wild-type or DUX-expressing ESC[5]. DUX-expressing ESC were sorted in GFP$^+$ or GFP$^-$ as a result of the activation of the LTR-GFP reporter[5]. Heatmap shows the top 50 most differentially expressed genes. Note that auxin-treated ESC$^{CTCF\text{-}AID}$ for four days still contain a high number of non-reprogrammed ESC. **d, e** HTI quantification of RFP$^+$ cells in untreated or auxin-treated for four days *LTR-RFP* reporter ESC$^{CTCF\text{-}AID}$ expressing DUX (**d**) or treated with a histone deacetylase inhibitor (**e**) where indicated. ESC$^{CTCF\text{-}AID}$ were infected with pCW57.1-mDUX-CA (Addgene 99284) and induced with 300ng/ml DOX. Center lines indicate mean values. n=2000; p values are shown from two-tailed unpaired *t*-tests. Percentages of RFP$^+$ cells above the threshold are indicated. **f,** Western blot analysis of the indicated proteins performed in two newly generated KH2-ESC$^{CTCF\text{-}AID}$ treated with auxin for 2 and 4 days. Parental ESC were used to show the smaller size and higher levels of CTCF. Tubulin levels are shown as a loading control. Two ESC cell lines are shown but 4 ESC lines from 2 different genetic backgrounds were generated and confirmed. **g,** HTI quantification of γH2AX in untreated or auxin-treated for four days in LTR-RFP reporter ESC$^{CTCF\text{-}AID}$. Auxin-treated cells were split based in RFP$^+$ and RFP$^-$. Center lines indicate mean values. n=500; p values are shown from two-tailed unpaired *t*-tests. For (**a**), (**b**), (**d**), (**e**), (**f**)

and (**g**), three (**a, b and g**) or two (**d, e and f**) independent experiments were performed with similar results but one representative experiment is shown.

Figure S8

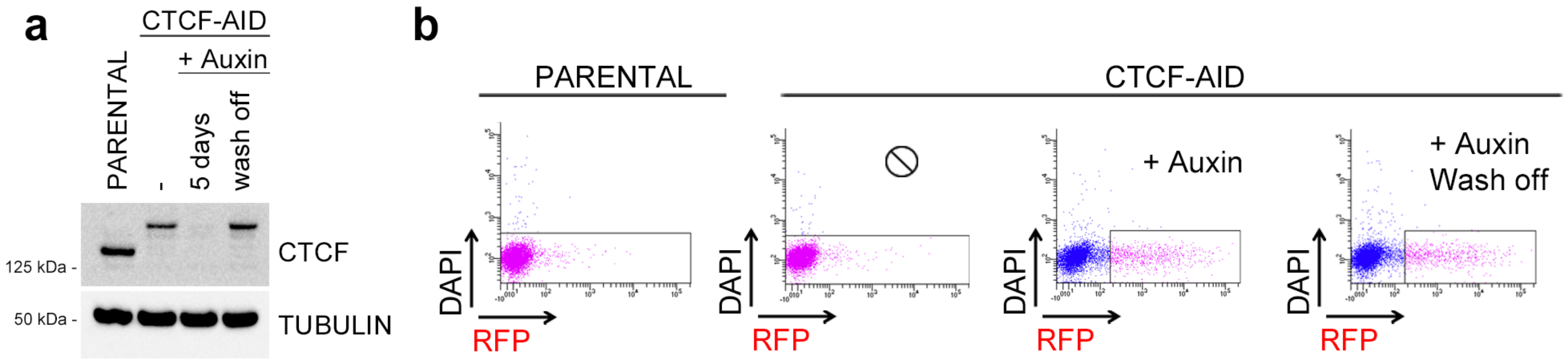
a
CTCF-AID
+ Auxin
PARENTAL
-
5 days
wash off
125 kDa -
CTCF
50 kDa -
TUBULIN
b
PARENTAL
CTCF-AID
+ Auxin
+ Auxin
Wash off
DAPI
RFP

Supplementary Fig. 8: Re-expression of CTCF promotes silencing of the transcriptional 2C-program. a, Western blot analysis of CTCF performed in untreated or auxin-treated for five days in parental ESC or $ESC^{CTCF\text{-}AID}$. Wash off sample include $ESC^{CTCF\text{-}AID}$ treated four days with auxin plus 18 hours without auxin. Tubulin levels are shown as a loading control. ∅=No treatment. **b,** Flow cytometry plots generated from samples described in (**a**). Gating shows the actual RFP^{+} cells sorted for the experiment. For (**a** and **b**) two independent experiments were performed with similar results but one representative is shown.

Figure S9

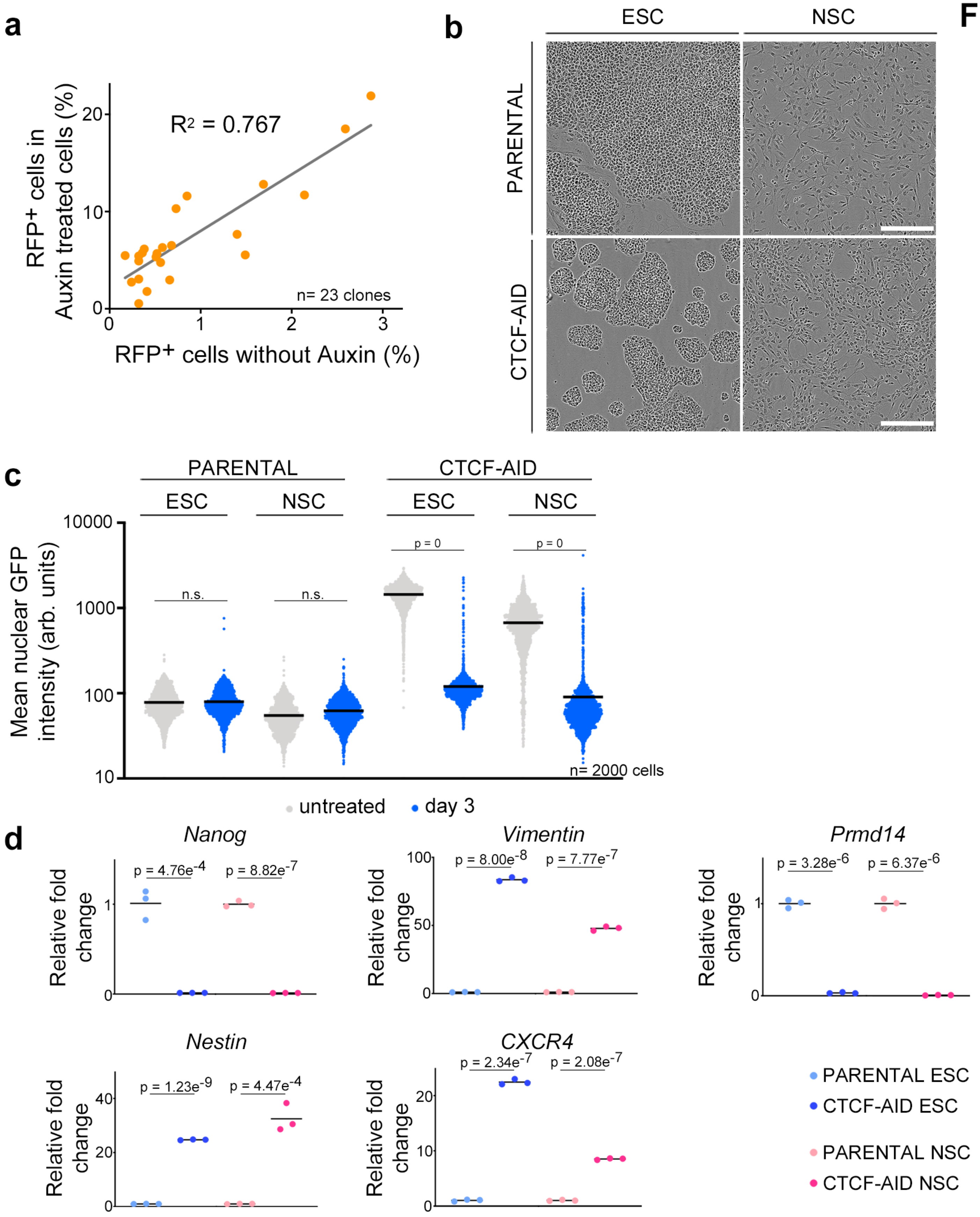

a
R2 = 0.767
RFP+ cells in Auxin treated cells (%)
RFP+ cells without Auxin (%)
n= 23 clones
b
ESC
NSC
PARENTAL
CTCF-AID
c
PARENTAL
CTCF-AID
ESC
NSC
ESC
NSC
Mean nuclear GFP intensity (arb. units)
n.s.
n.s.
p = 0
p = 0
n= 2000 cells
untreated
day 3
d
Nanog
p = 4.76e-4
p = 8.82e-7
Vimentin
p = 8.00e-8
p = 7.77e-7
Prmd14
p = 3.28e-6
p = 6.37e-6
Nestin
p = 1.23e-9
p = 4.47e-4
CXCR4
p = 2.34e-7
p = 2.08e-7
Relative fold change
PARENTAL ESC
CTCF-AID ESC
PARENTAL NSC
CTCF-AID NSC

Supplementary Fig. 9: Epigenetic and/or transcriptional roadblocks prevent 2C-like conversion in CTCF-depleted ESC$^{CTCF-AID}$. a, Graph showing the percentage of endogenous 2C-like cells in a total of 23 subclonal ESC lines derived from the parental ESC$^{CTCF-AID}$. Cells were untreated or auxin-treated for four days. A linear regression analysis was performed, and the regression coefficient (R) is shown. **b,** Representative bright-field images from paraformaldehyde-fixed ESC, NSC, ESC$^{CTCF-AID}$ and NSC$^{CTCF-AID}$. Scale bar, 200μm. **c,** HTI quantification of GFP in ESC, NSC, ESC$^{CTCF-AID}$ and NSC$^{CTCF-AID}$ untreated or treated with auxin for four days. Center lines indicate mean values. p values are shown from two-tailed unpaired *t*-tests. n.s.= non-significant. Three independent experiments were performed but one representative is shown. **d,** Graphs showing the relative fold change (log2) expression of pluripotent genes expressed in ESC (*Nanog* and *Prdm14*) and NSC associated markers (*Cxcr4*, *Nestin* and *Vimentin*) in ESC, NSC, ESC$^{CTCF-AID}$ and NSC$^{CTCF-AID}$. p values are shown from two-tailed unpaired *t*-tests. Center line indicates the mean. Reactions were performed by triplicate in two independent experiments.

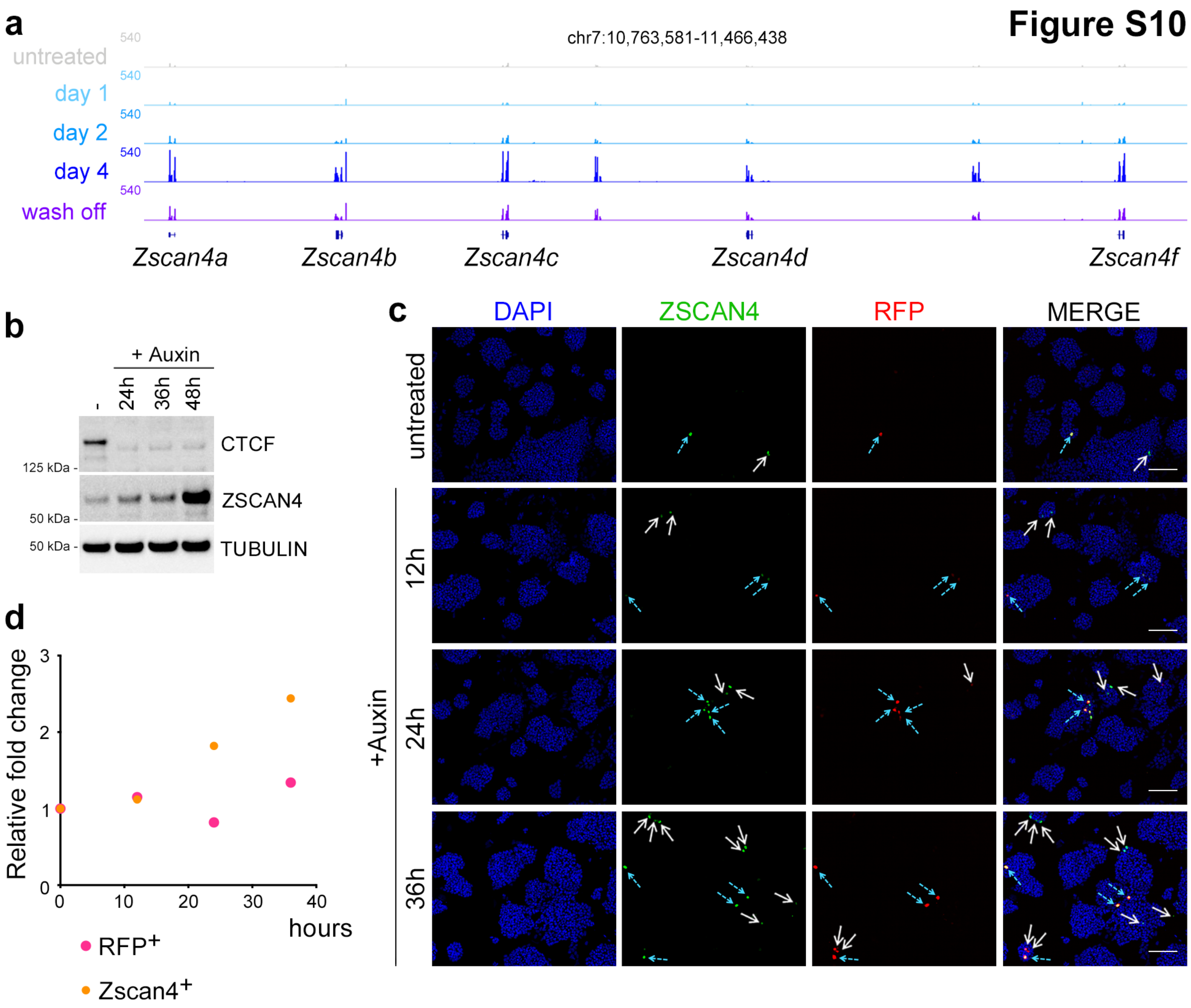

Figure S10
a
chr7:10,763,581-11,466,438
untreated
day 1
day 2
day 4
wash off
540
Zscan4a
Zscan4b
Zscan4c
Zscan4d
Zscan4f
b
+ Auxin
-
24h
36h
48h
CTCF
ZSCAN4
TUBULIN
125 kDa -
50 kDa -
50 kDa -
c
DAPI
ZSCAN4
RFP
MERGE
untreated
+Auxin
12h
24h
36h
d
Relative fold change
hours
0
1
2
3
10
20
30
40
RFP+
Zscan4+

Supplementary Fig. 10: ZSCAN4 expression is necessary for efficient 2C-like conversion upon CTCF depletion. a, Genome browser tracks showing RNAseq RPKM read count at the ZSCAN4 cluster in the indicated samples. **b,** Western blot analysis of CTCF and ZSCAN4 performed in untreated or auxin-treated for the indicated hours in $ESC^{CTCF\text{-}AID}$. Tubulin levels are shown as a loading control. **c,** Representative immunofluorescence images from $ESC^{CTCF\text{-}AID}$ treated with auxin for one or two days showing $ZSCAN4^{+}$ cells and $ZSCAN4^{+}$; $LTR\text{-}RFP^{+}$ cells. Scale bar, 200μm. White arrows indicate single $ZSCAN4^{+}$ or $LTR\text{-}RFP^{+}$ cells. Blue dashed arrows indicate double $ZSCAN4^{+}$; $LTR\text{-}RFP^{+}$ cells**. d,** Graph showing the relative fold-change in the number of $ZSCAN4^{+}$ or $LTR\text{-}RFP^{+}$ cells related to time=0 (untreated $ESC^{CTCF\text{-}AID}$) at the indicated timepoints (12, 24 and 36 hours) after the addition of auxin. Average value from triplicates is shown. In (**b-d**) two independent experiments were performed but only one representative is shown.

Figure S11

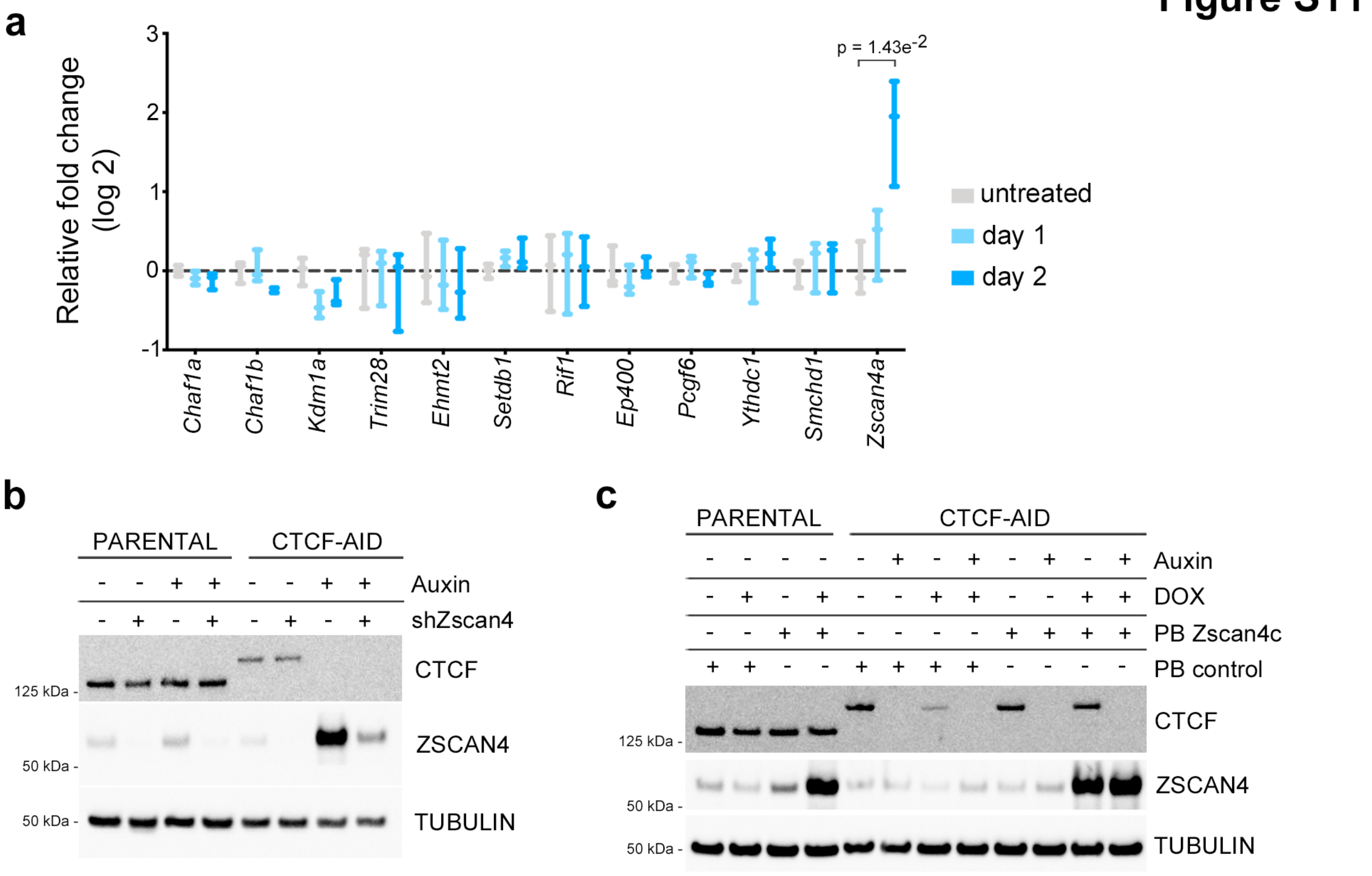

a
Relative fold change (log 2)
3
2
1
0
-1
p = 1.43e-2
untreated
day 1
day 2
Chaf1a
Chaf1b
Kdm1a
Trim28
Ehmt2
Setdb1
Rif1
Ep400
Pcgf6
Ythdc1
Smchd1
Zscan4a
b
PARENTAL
CTCF-AID
Auxin
shZscan4
CTCF
ZSCAN4
TUBULIN
125 kDa -
50 kDa -
50 kDa -
c
PARENTAL
CTCF-AID
Auxin
DOX
PB Zscan4c
PB control
CTCF
ZSCAN4
TUBULIN
125 kDa -
50 kDa -
50 kDa -

Supplementary Fig. 11:

a, Graph showing the relative fold change (log2) in the expression of the indicated known regulators involved in 2C-like conversion in $ESC^{CTCF\text{-}AID}$ untreated or auxin-treated for one or two days. Three independent values per gene are shown. Data was obtained from RNAseq datasets[27]. p value is shown from two-tailed unpaired *t*-test. **b,** Western blot analysis of CTCF and ZSCAN4 performed in untreated or auxin-treated for four days in parental ESC or $ESC^{CTCF\text{-}AID}$. ESC were infected with lentiviruses expressing siRNAs against ZSCAN4 where indicated. Tubulin levels are shown as a loading control. **c,** Western blot analysis of CTCF and ZSCAN4 performed in untreated or auxin-treated for four days in parental ESC or $ESC^{CTCF\text{-}AID}$. ESC were transfected with PiggyBac (PB) control or expressing ZSCAN4 where indicated. 1μg/ml DOX was added to induce PB-induced expression. Tubulin levels are shown as a loading control. For (**b** and **c**) two independent experiments with similar results were performed but only one representative is shown.

Supplementary Table 1: List of primers.

**Primers (qPCR)**		
	forward	reverse
DUX	GGAGTGAGAGGCAGATCAGG	CTGCTGACCGAAGTCCAACT
ZSCAN4C	TCTTTCTGGTTGGCAGCTTT	GCCAGGCTTCTGTCAAGAAC
ZFP352	AAGGTCCCACATCTGAAGAA	GGGTATGAGGATTCACCCA
TCSTV3	ACCAGCTGAAACATCCATCC	CCATGGATCCCTGAAGGTAA
SP110	CACCTGCAAACAAGAAAGCA	AACTCCATGTCCAGGTGAGG
TDPOZ1	GCCCTTGATTTCATTGCCTA	CACTTTCGGCTCCAAGAAAG
DUB1	GCCTTCAAGTGCACAGACAA	GATGTCCAGGAAGCGATCAT
EIF1Ad8 (GM8300)	AACAGGCGCAGAGGTAAAAA	GCACAGCCTCCTTACACCAT
NANOG	CAGGTGTTTGAGGGTAGCTC	CGGTTCATCATGGTACAGTC
PRDM14	CACTCCCGAAGTACCACGAT	CCCACCTCTGACCACTGATT
VIMENTIN	TGAAGGAAGAGATGGCTCGT	TCCAAGCAGCTTCCTGTAGGT
NESTIN	CTCGAGCAGGAAGTGGTAGG	GCCTCTTTTGGTTCCTTTCC
CXCR4	GGGTCATCAAGCAAGGATGT	GGCAGAGCTTTTGAACTTGG
**Primers (Cloning)**		
LTR-NotI-F (Cloning LTR sequences into the PiggyBac construct)	CGATGCGGCCGCGCTGTAGTGGTTATTCCTGGTTGTC	
LTR-EcoRI-R (Cloning LTR sequences into the PiggyBac construct)	CGGAGAATTCAAGCTTTGTGCACTGAATCACCT	
mDUX-FLAG-MfeI-F (Cloning DUX into pBS31)	GGCATCAATTGACCATGGACTACAAGGATGACGACGATAAGGGCAGCGGAGCTGAG GCTGGCTCTCCAGTGGGAGGAT	
mDUX-FLAG-MfeI-R (Cloning DUX into pBS31)	GGCATCAATTGTTACAGCATGTCAAGAAGGGTCTGGTACTC	
