# Supplementary material for: CTCF is a barrier for 2C-like reprogramming
Source: Nat Commun. 2021 Aug 11;12:4856. doi: 10.1038/s41467-021-25072-x (PMC8358036; doi:10.1038/s41467-021-25072-x)
Supplement: Supplementary file 3 — Reporting Summary [file 41467_2021_25072_MOESM3_ESM.pdf]

Corresponding author(s): Sergio Ruiz

Last updated by author(s): July 1, 2021

Reporting Summary

Nature Research wishes to improve the reproducibility of the work that we publish. This form provides structure for consistency and transparency in reporting. For further information on Nature Research policies, see our Editorial Policies and the Editorial Policy Checklist.

Statistics

For all statistical analyses, confirm that the following items are present in the figure legend, table legend, main text, or Methods section.

n/a	Confirmed	
☐	☒	The exact sample size (*n*) for each experimental group/condition, given as a discrete number and unit of measurement
☐	☒	A statement on whether measurements were taken from distinct samples or whether the same sample was measured repeatedly
☐	☒	The statistical test(s) used AND whether they are one- or two-sided<br>*Only common tests should be described solely by name; describe more complex techniques in the Methods section.*
☒	☐	A description of all covariates tested
☒	☐	A description of any assumptions or corrections, such as tests of normality and adjustment for multiple comparisons
☐	☒	A full description of the statistical parameters including central tendency (e.g. means) or other basic estimates (e.g. regression coefficient) AND variation (e.g. standard deviation) or associated estimates of uncertainty (e.g. confidence intervals)
☐	☒	For null hypothesis testing, the test statistic (e.g. *F*, *t*, *r*) with confidence intervals, effect sizes, degrees of freedom and *P* value noted<br>*Give P values as exact values whenever suitable.*
☒	☐	For Bayesian analysis, information on the choice of priors and Markov chain Monte Carlo settings
☒	☐	For hierarchical and complex designs, identification of the appropriate level for tests and full reporting of outcomes
☐	☒	Estimates of effect sizes (e.g. Cohen's *d*, Pearson's *r*), indicating how they were calculated

Our web collection on statistics for biologists contains articles on many of the points above.

Software and code

Policy information about availability of computer code

Data collection	Detailed in the manuscript.
Data analysis	Detailed in the manuscript. The software we used to process Cut&Run data is a reimplementation of a published pipeline (https://genomebiology.biomedcentral.com/articles/10.1186/s13059-019-1802-4) so that that we can more easily run it on our NIH high-performance compute cluster or DNANexus. Nothing novel was done, and all processing steps were run using established bioinformatics software. If we changed any parameters in the pipeline, it was noted the methods. Here, we include the code for the pipeline in the in the attached .zip, which contains instructions for its use and is also available here: https://github.com/desireetillo/CTCF_CutAndRun21. An example input/outputs can be found in this link: https://hpc.nih.gov/~tillodc/CandR_example.zip.

For manuscripts utilizing custom algorithms or software that are central to the research but not yet described in published literature, software must be made available to editors and reviewers. We strongly encourage code deposition in a community repository (e.g. GitHub). See the Nature Research guidelines for submitting code & software for further information.

Data

Policy information about availability of data

All manuscripts must include a data availability statement. This statement should provide the following information, where applicable:

- Accession codes, unique identifiers, or web links for publicly available datasets
- A list of figures that have associated raw data
- A description of any restrictions on data availability

All data supporting the findings of this study are available within the paper and its supplementary information file. Source data are provided with this paper. All sequencing data are available through Gene Expression Omnibus accession number: GSE165162.

Field-specific reporting

Please select the one below that is the best fit for your research. If you are not sure, read the appropriate sections before making your selection.

[x] Life sciences [ ] Behavioural & social sciences [ ] Ecological, evolutionary & environmental sciences

For a reference copy of the document with all sections, see nature.com/documents/nr-reporting-summary-flat.pdf

Life sciences study design

All studies must disclose on these points even when the disclosure is negative.

Sample size	Number of independent cellular and molecular biology experiments and replicates are carefully described in Methods and Figure legends. Next-generation sequencing experiments (ENDseq, ChIPseq and Cut&Run) were performed in two independent ESC lines which is sufficient to determine reproducible results based on extensive experience and is widely accepted.
Data exclusions	No data were excluded from the analyses.
Replication	All results were reliably reproduced in multiple independent experiments as indicated in the figure legends.
Randomization	Randomization was not necessary for our study as it does not involve a case-control cohort experiment.
Blinding	All mouse embryos in the study were obtained from wild-type mice and thus there was no need to examine the embryos blindly.

Reporting for specific materials, systems and methods

We require information from authors about some types of materials, experimental systems and methods used in many studies. Here, indicate whether each material, system or method listed is relevant to your study. If you are not sure if a list item applies to your research, read the appropriate section before selecting a response.

Materials & experimental systems

n/a	Involved in the study
[ ]	[x] Antibodies
[ ]	[x] Eukaryotic cell lines
[x]	[ ] Palaeontology and archaeology
[ ]	[x] Animals and other organisms
[x]	[ ] Human research participants
[x]	[ ] Clinical data
[x]	[ ] Dual use research of concern

Methods

n/a	Involved in the study
[ ]	[x] ChIP-seq
[ ]	[x] Flow cytometry
[x]	[ ] MRI-based neuroimaging

Antibodies

Antibodies used

Antibodies used for western blot: p-KAP1 (dilution 1:1000, A300-767A, Bethyl, RRID:AB_669740) or ZSCAN4C (1:500, AB4340, Millipore Sigma, RRID:AB_2827621), γH2AX, (1:1000, 05-636, Millipore, RRID:AB_309864), CTCF (1:1000, 07-729, Millipore, RRID:AB_441965), Flag (1:1000, F1804, Sigma Aldrich, RRID:AB_262044), Tubulin (1:50000, T9026, Sigma-Aldrich, RRID:AB_477593), Goat anti-Rabbit IgG (H+L) Secondary Antibody, HRP (1:5000; Thermo Fisher Scientific, Cat# 31466, RRID:AB_10960844); Goat anti-Mouse IgG (H+L) Secondary Antibody, HRP (1:5000; Thermo Fisher Scientific, Cat# 31431, RRID:AB_10960845).

Antibodies used for immunofluorescence: OCT3/4 (1:100, sc-5279, Santa Cruz Biotechnology, RRID:AB_628051), ZSCAN4 (1:2000, AB4340, Millipore Sigma, RRID:AB_2827621), γH2AX (1:1000, 05-636, Millipore, RRID:AB_309864), CTCF (1:1000, ab188408, Abcam, RRID:AB_2819212), Flag (1:500, F1804, Sigma Aldrich, RRID:AB_262044).

Antibodies used for Cut&Run (2ug): CTCF (07-729, Millipore, RRID:AB_441965), Guinea Pig anti-Rabbit IgG (ABIN101961, Antibodies-online, RRID:AB_10775589).
Antibodies used for ChIPseq (10ug): anti-RPA32 antibody (Abcam ab10359, RRID:AB_297095), Guinea Pig anti-Rabbit IgG (ABIN101961, Antibodies-online, , RRID:AB_10775589).

Secondary Antibodies used for IF: Chicken anti-Rabbit IgG (H+L) Cross-Adsorbed Secondary Antibody, Alexa Fluor 488 (Thermo Fisher Scientific, Cat# 31431, RRID:AB_10960845), Goat anti-Mouse IgG (H+L) Cross-Adsorbed Secondary Antibody, Alexa Fluor 488 (Thermo Fisher Scientific, Cat# A-11001, RRID:AB_2534069), Chicken anti-Rabbit IgG (H+L) Cross-Adsorbed Secondary Antibody, Alexa Fluor 647 (Thermo Fisher Scientific, Cat# A-21443, RRID:AB_2535861), Chicken anti-Mouse IgG (H+L) Cross-Adsorbed Secondary Antibody, Alexa Fluor 647 (Thermo Fisher Scientific, Cat# A-21463, RRID:AB_2535869)

Validation

The antibodies used in this work are all commercial and widely tested in literature for the proposed assay (see below).

Antibodies used for western blot:

p-KAP1 (dilution 1:1000, A300-767A, Bethyl, RRID:AB_669740)
53BP1 inhibits homologous recombination in Brca1-deficient cells by blocking resection of DNA breaks
Bunting et al., 2010. DOI: 10.1016/j.cell.2010.03.012

ZSCAN4C (1:500, AB4340, Millipore Sigma, RRID:AB_2827621)
TRF2-mediated telomere protection is dispensable in pluripotent stem cells
Markiewicz-Potoczny et al., 2021. DOI: 10.1038/s41586-020-2959-4

γH2AX, (1:1000, 05-636, Millipore, RRID:AB_309864)
Limiting replication stress during somatic cell reprogramming reduces genomic instability in induced pluripotent stem cells
Ruiz et al., 2015. DOI: 10.1038/ncomms9036

CTCF (1:1000, 07-729, Millipore, RRID:AB_441965)
Genome-wide targeting of the epigenetic regulatory protein CTCF to gene promoters by the transcription factor TFII-I
Peña-Hernández et al., 2015. DOI: 10.1073/pnas.1416674112

Flag (1:1000, F1804, Sigma Aldrich, RRID:AB_262044)
A unique feature of swine ANP32A provides susceptibility to avian influenza virus infection in pigs
Zhang et al., 2020. DOI: 10.1371/journal.ppat.1008330

Tubulin (1:50000, T9026, Sigma-Aldrich, RRI<D:AB_477593)
Limiting replication stress during somatic cell reprogramming reduces genomic instability in induced pluripotent stem cells
Ruiz et al., 2015. DOI: 10.1038/ncomms9036

Antibodies used for immunofluorescence:

OCT3/4 (1:100, sc-5279, Santa Cruz Biotechnology, RRID:AB_628051)
TRF2-mediated telomere protection is dispensable in pluripotent stem cells
Markiewicz-Potoczny et al., 2021. DOI: 10.1038/s41586-020-2959-4

ZSCAN4 (1:2000, AB4340, Millipore Sigma, RRID:AB_2827621)
TRF2-mediated telomere protection is dispensable in pluripotent stem cells
Markiewicz-Potoczny et al., 2021. DOI: 10.1038/s41586-020-2959-4

γH2AX (1:1000, 05-636, Millipore, RRID:AB_309864)
Limiting replication stress during somatic cell reprogramming reduces genomic instability in induced pluripotent stem cells
Ruiz et al., 2015. DOI: 10.1038/ncomms9036

CTCF (1:1000, ab188408, Abcam, RRID:AB_2819212).
This antibody has been vallidated for multiple applications including ChIP-sequencing, CUT&Tag-seq, IHC-P, ChIP, ICC/IF and WB. For immunofluorescence analysis, validation was performed using Hela and NIH/3T3 cells. https://www.abcam.com/ctcf-antibody-epr18253-chip-grade-ab188408.html

Flag (1:500, F1804, Sigma Aldrich, RRID:AB_262044).
CRISPR-Cas9-Mediated Epitope Tagging Provides Accurate and Versatile Assessment of Myocardin-Brief Report
Lyu et al, 2018. DOI: 10.1161/ATVBAHA.118.311171

Antibodies used for Cut&Run (2ug):

CTCF (07-729, Millipore, RRID:AB_441965)
Single-cell CUT&Tag analysis of chromatin modifications in differentiation and tumor progression
Wu et al., 2021. DOI: 10.1038/s41587-021-00865-z

Guinea Pig anti-Rabbit IgG (ABIN101961, Antibodies-online, RRID:AB_10775589)
Single-cell CUT&Tag analysis of chromatin modifications in differentiation and tumor progression
Wu et al., 2021. DOI: 10.1038/s41587-021-00865-z

Antibodies used for ChIPseq (10ug):

anti-RPA32 antibody (Abcam ab10359, RRID:AB_297095)
Dual Roles of Poly(dA:dT) Tracts in Replication Initiation and Fork Collapse
Tubbs et al., 2018. DOI: 10.1016/j.cell.2018.07.011

Guinea Pig anti-Rabbit IgG (ABIN101961, Antibodies-online, , RRID:AB_10775589)
Single-cell CUT&Tag analysis of chromatin modifications in differentiation and tumor progression
Wu et al., 2021. DOI: 10.1038/s41587-021-00865-z

Eukaryotic cell lines

Policy information about cell lines

Cell line source(s)	293T (ATCC), R1 ES cells, G4 ES cells and KH2 ES cells (were a gift from Sagrario Ortega, Transgenic mouse Unit, CNIO, Madrid), E14 ESC, EN52.9.1 ES cells and EN204.3 ES cells (were a gift from Benoit Bruneau (Gladstone Institute of Cardiovascular Disease, San Francisco) and Elphege Nora (Department of Biochemistry and Biophysics, University of California San Francisco, San Francisco)).
Authentication	None of the cell lines used have been authenticated.
Mycoplasma contamination	All cell lines were confirmed negative to mycoplasma contamination
Commonly misidentified lines (See ICLAC register)	None of the cell lines used in this manuscript are listed in the ICLAC Database of Cross-contaminated or Misidentified Cell Lines.

Animals and other organisms

Policy information about studies involving animals; ARRIVE guidelines recommended for reporting animal research

Laboratory animals	Mouse embryos were obtained from the mating of 4 weeks-old males and females. C57BL6 background.
Wild animals	Our study did not involve wild animals.
Field-collected samples	Our study did not involve field-collected samples.
Ethics oversight	All mouse breeding and experimentation followed protocols approved by the National Institutes of Health Institutional Animal Care and use committee. Mice were maintained in a dark/light cycle of 12 hours each in a temperature range of 68o-76oF and a range of 30-70% humidity.

Note that full information on the approval of the study protocol must also be provided in the manuscript.

ChIP-seq

Data deposition

- [x] Confirm that both raw and final processed data have been deposited in a public database such as GEO.
- [x] Confirm that you have deposited or provided access to graph files (e.g. BED files) for the called peaks.

Data access links
May remain private before publication.

https://www.ncbi.nlm.nih.gov/geo/query/acc.cgi?acc=GSE165162

Files in database submission

The following files correspond to Cut&Run experiments:

ESC_DUX_Clone 1_ DOX_16h_RFPneg_CTCF (GSM5027345)
ESC_DUX_Clone 1_ DOX_16h_RFPpos_CTCF (GSM5027346)
ESC_DUX_Clone 1_ DOX_24h_RFPneg_CTCF) (GSM5027347)
ESC_DUX_Clone 1_ DOX_24h_RFPpos_CTCF (GSM5027348)
ESC_DUX_Clone 1_CTCF (GSM5027349)
ESC_DUX_Clone 1_IgG (GSM5027350)
ESC_DUX_Clone 2_ DOX_16h_RFPneg_CTCF (GSM5027351)
ESC_DUX_Clone 2_ DOX_16h_RFPpos_CTCF (GSM5027352)
ESC_DUX_Clone 2_ DOX_24h_RFPneg_CTCF (GSM5027353)
ESC_DUX_Clone 2_ DOX_24h_RFPpos_CTCF (GSM5027354)
ESC_DUX_Clone 2_CTCF (GSM5027355)
ESC_DUX_Clone 2_IgG (GSM5027356)
E14_RFPneg_CTCF (GSM5027357)
E14_IgG (GSM5027358)
E14_RFPpos_CTCF (GSM5027359)
R1_RFPneg_CTCF (GSM5027360)
R1_IgG (GSM5027361)

R1_RFPpos_CTCF (GSM5027362)

The following files correspond to ChIPseq experiments:

ESC_DUX_Clone1_IgG_ChIP (GSM5241374)
ESC_DUX_Clone1_RPA_ChIP (GSM5241375)
ESC_DUX_Clone1_DOX_16h_RPA_ChIP (GSM5241376)
ESC_DUX_Clone2_IgG_ChIP (GSM5241377)
ESC_DUX_Clone2_RPA_ChIP (GSM5241378)
ESC_DUX_Clone2_DOX_16h_RPA_ChIP (GSM5241379)

Genome browser session (e.g. UCSC): N/A

Methodology

Replicates: At least 2 replicates were performed.

Sequencing depth: For Cut&Run experiments, paired-end sequencing (75 bp) at 10-15 million reads per sample were at least sequenced as recommended in previous publications. For ChIPseq experiments, single-end sequencing (75 bp) at 25 million reads per sample were at least sequenced as recommended in previous publications.

Antibodies: All antibodies and sources are provided in the Methods section.

Peak calling parameters: Parameters are specified in the Methods section.

Data quality: Data quality parameters are specified in the Methods section.

Software: All software used described in the Methods section.

Flow Cytometry

Plots

Confirm that:

- [x] The axis labels state the marker and fluorochrome used (e.g. CD4-FITC).
- [x] The axis scales are clearly visible. Include numbers along axes only for bottom left plot of group (a 'group' is an analysis of identical markers).
- [ ] All plots are contour plots with outliers or pseudocolor plots.
- [x] A numerical value for number of cells or percentage (with statistics) is provided.

Methodology

Sample preparation: For live cell flow cytometry experiments, cells were dissociated into single cell suspensions and analyzed for RFP expression, DAPI was added to detect cells with compromised membrane integrity. For EdU Click-IT experiments, cells were incubated for 20 min with 10 µM EdU, fixed in 4 % paraformaldehyde, permeabilized in 0.5 % triton X-100, followed by Alexa Flour 488-azide or Alexa Flour 647-azide Click-iT labeling chemistry. DNA content was stained using DAPI or Hoechst 33342 (62249, Thermo Fisher Scientific).

Instrument: LSRFortessa (BD Biosciences), BD FACSAria Fusion and FACSymphony A5 instrument (BD Biosciences)

Software: FlowJo Version 10.7.1.

Cell population abundance: An small portion of the sorted cells were re-analyzed by Flow Cytometry again in the BD FACSAria Fusion to confirm a purity of over 95%.

Gating strategy: Gating was performed as follows: FSC/SSC -> Singlets -> Live cells (DAPI negative) -> gating of interest. The appropriate negative control was used for generating the gates of interest. For instance, LTR_RFP positive cells were identified by using control ESC without the reporter.

- [x] Tick this box to confirm that a figure exemplifying the gating strategy is provided in the Supplementary Information.
