# Supplementary material for: CTCF is a barrier for 2C-like reprogramming
Source: Nat Commun. 2021 Aug 11;12:4856. doi: 10.1038/s41467-021-25072-x (PMC8358036; doi:10.1038/s41467-021-25072-x)
Supplement: Supplementary file 4 — Description of Additional Supplementary Files [file 41467_2021_25072_MOESM4_ESM.pdf]

Description of Additional Supplementary Files

File Name: Supplementary Data 1
Description: File containing the set of overlapping ENDseq peaks identified in the ESC^{Dux} Clone 1 and Clone 2 used in this manuscript.

File Name: Supplementary Data 2
Description: File containing the set of ENDseq peaks identified in the ESC^{Dux} Clone 1 used in this manuscript.

File Name: Supplementary Data 3
Description: File containing the set of ENDseq peaks identified in the ESC^{Dux} Clone 2 used in this manuscript.

File Name: Supplementary Data 4
Description: File containing the set of genes associated by proximity to the set of overlapping ENDseq peaks identified in the ESC^{Dux} Clone 1 and Clone 2 used in this manuscript.

File Name: Supplementary Data 5
Description: File containing the set of upregulated or downregulated genes upon DUX expression associated by proximity to the set of overlapping ENDseq peaks identified in the ESC^{Dux} Clone 1 and Clone 2 used in this manuscript.

File Name: Supplementary Data 6
Description: File containing the set of overlapping ENDseq peaks identified in the ESC^{Dux} Clone 1 and Clone 2 used in this manuscript enriched in CTCF binding.

File Name: Supplementary Data 7
Description: File containing the different sets of CTCF peaks identified in untreated and DOX-treated sorted RFPneg or sorted RFPpos cells obtained from the LTR-RFP reporter ESC^{Dux} Clone 1 and Clone 2 used in this manuscript.

File Name: Supplementary Data 8
Description: File containing the different sets of CTCF peaks identified in sorted RFPneg or sorted RFPpos cells from the wild-type LTR-RFP reporter ESC WT1 and WT2 used in this manuscript.

File Name: Supplementary Movie 1
Description: Live cell imaging of DOX-treated LTR-RFP reporter ESC^{Dux} expressing H2B-GFP.

File Name: Supplementary Movie 2
Description: Live cell imaging of LTR-RFP reporter wild-type ESC expressing H2B-GFP.

File Name: Supplementary Movie 3
Description: Live cell imaging of LTR-RFP reporter $ESC^{CTCF-AID}$ upon auxin addition.

File Name: Supplementary Software 1
Description: Codes for the pipeline used to analyze the Cut&Run data from this manuscript.
